# Simultaneous Implant Placement and Guided Bone Regeneration Using Freeze-Dried Bone Allograft and Amnion–Chorion Membrane for Post-traumatic Anterior Maxillary Bone Dehiscence: A Case Report

**DOI:** 10.7759/cureus.115497

**Published:** 2026-08-31

**Authors:** Aena J Pundir, Harsha P Tembhare, Siddharth Pundhir, Prajna N Behuria, Shivani Sayankar

**Affiliations:** 1 Periodontics, Rungta Dental College and Hospital, Rungta, IND; 2 Periodontics, Rungta College of Dental Science and Research, Bhilai, IND; 3 Oral Pathology and Microbiology, Rungta College of Dental Science and Research, Bhillai, IND; 4 Periodontology, Rungta College of Dental Science and Research, Bhilai, IND

**Keywords:** aesthetic outcome, amnion-chorion membrane, bone dehiscence, guided bone regeneration, implants

## Abstract

Implant placement in the aesthetic zone is challenging due to enhanced patient demands and potential bone deficiencies. Bone loss in this area is often due to trauma and periodontal disease, which complicates implant placement. A patient presented with missing upper anterior teeth and significant bone loss following trauma from a road accident. Clinical and radiographic evaluations were carried out using cone-beam computed tomography (CBCT) that indicated bone dehiscence and inadequate bone for implant. After a thorough case study, a treatment plan was made, involving placement of dental implants with simultaneous guided bone regeneration around the implants using freeze-dried bone allograft (FDBA) and amnion chorion membrane to enhance osseointegration and restore the ridge, thereby supporting the patient’s functional and aesthetic needs. Bone grafting using FDBA combined with amnion chorion membrane around implants can be successfully employed in cases of bone dehiscence. Simultaneously, meticulous planning and substantial execution can provide promising results.

## Introduction

Implant rehabilitation in the anterior maxilla presents a particular clinical challenge because treatment must restore both function and the three-dimensional architecture required for an acceptable esthetic outcome. The anterior maxillary region is highly susceptible to alveolar bone loss following traumatic injuries, periodontal disease, and tooth extraction. Loss of the facial cortical plate may result in horizontal and/or vertical ridge deficiencies, which can compromise ideal implant positioning and subsequently affect peri-implant tissue contours, prosthetic emergence profile, and long-term esthetic stability [1,2]. Therefore, adequate hard-tissue volume is essential for placement of an implant in a prosthetically driven position and for predictable peri-implant tissue health.

Guided bone regeneration (GBR) is a commonly used regenerative approach for the management of localized alveolar ridge deficiencies. The technique involves placement of a barrier membrane that maintains a protected space over the osseous defect, limiting the migration of rapidly proliferating soft-tissue cells and allowing bone-forming cells to repopulate the defect [3]. GBR may be performed either before implant placement as a staged procedure or simultaneously with implant placement when adequate primary implant stability can be achieved. Evidence from clinical studies has demonstrated the applicability of GBR for correction of peri-implant dehiscence and fenestration defects and for augmentation of deficient alveolar ridges [3-5].

The choice of grafting material and barrier membrane is an important component of GBR because the biological and handling characteristics of these materials influence space maintenance, graft stability, and tissue regeneration. Bone-grafting materials used in implant-site development include autogenous bone, allografts, xenografts, and synthetic substitutes, with each material exhibiting different biological and physical properties [6]. Freeze-dried bone allograft (FDBA) is an allogeneic graft material primarily used for its osteoconductive scaffold function, providing a framework that can support the formation of new bone within a deficient site [7]. In addition to the graft material, selection of an appropriate barrier membrane is important for maintaining compartmentalization of the regenerative site. Amnion-chorion membranes have been investigated as biologically derived barrier membranes because of their favorable handling characteristics and reported properties relevant to soft-tissue healing and periodontal/peri-implant regeneration [8,9]. However, the clinical effectiveness of these membranes should be interpreted in the context of the available evidence, as the quality and extent of clinical evidence remain variable.

Simultaneous implant placement with GBR can be advantageous in appropriately selected cases because it allows implant positioning and ridge augmentation to be performed during the same surgical intervention, potentially reducing treatment time and avoiding a separate implant-placement procedure [5]. However, this approach requires careful three-dimensional treatment planning, adequate primary implant stability, appropriate management of the osseous defect, graft stabilization, and tension-free primary wound closure. In extensive defects, the decision between simultaneous and staged augmentation should be based on the morphology of the defect, residual bone volume, implant stability, and the ability to achieve predictable regenerative conditions.

In the present case, a post-traumatic anterior maxillary ridge defect with significant facial bone dehiscence was managed using simultaneous implant placement and GBR with FDBA and an amnion-chorion membrane. Preoperative clinical and cone-beam computed tomography (CBCT) assessment demonstrated inadequate facial bone volume for ideal implant positioning. The case describes the diagnostic evaluation, treatment planning, surgical management, and subsequent healing and implant-uncovering phases, highlighting the clinical considerations involved in managing a compromised anterior maxillary implant site.

## Case presentation

A 21-year-old female patient presented with missing teeth in the anterior maxillary region following a road traffic accident, associated with substantial loss of the alveolar ridge. Clinical examination revealed a localized deficiency of the anterior maxillary ridge with reduced buccolingual dimensions and loss of the facial cortical plate, resulting in bone dehiscence at the proposed implant sites. The surrounding soft tissues were clinically healthy, with no signs of active infection or inflammation. The adjacent teeth and periodontal tissues were evaluated to determine the suitability of the planned implant sites (Figure 1).

**Figure 1:**
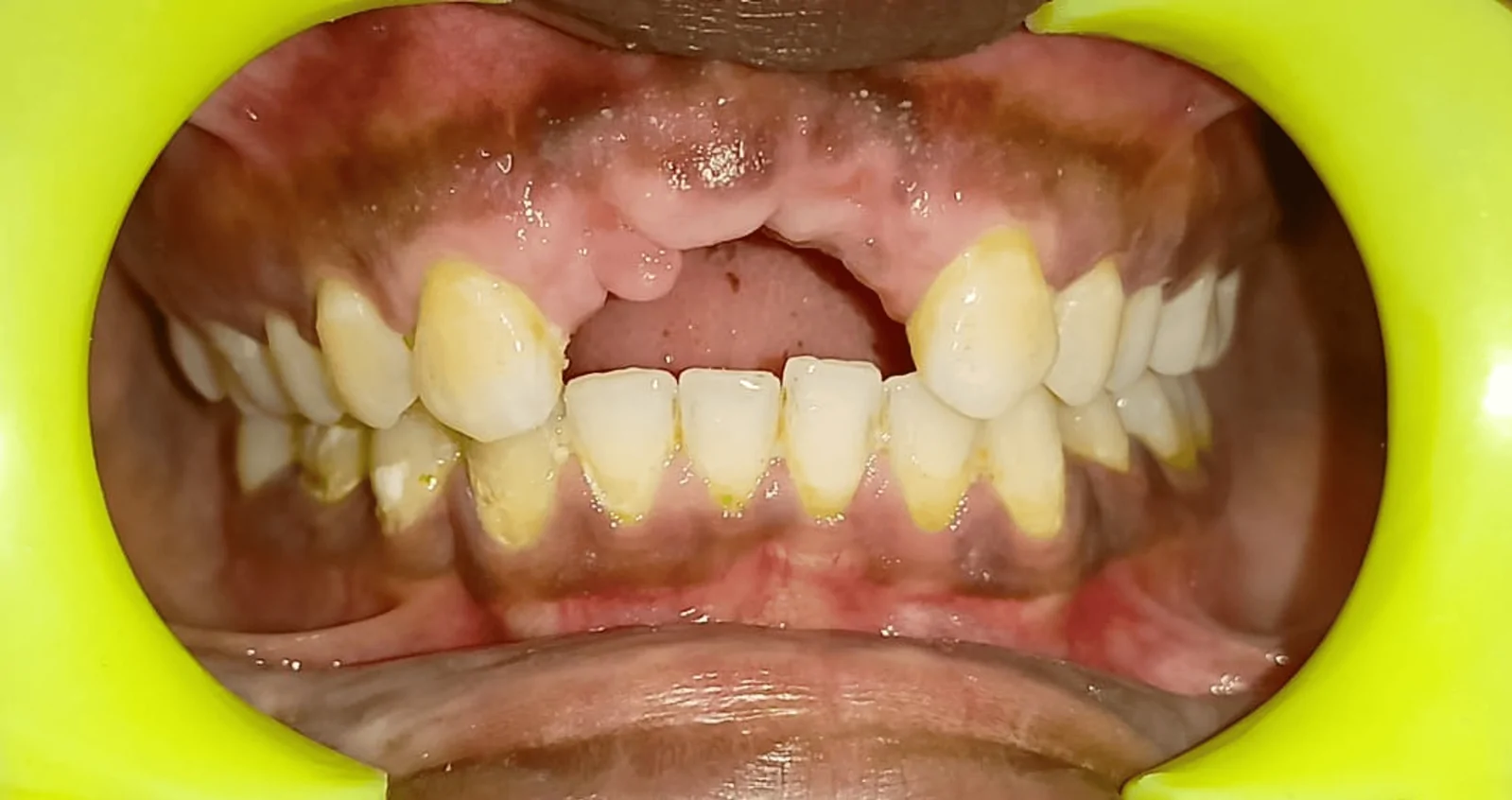
Pre operative clinical picture showing extensive ridge deficiency in anterior region

Cone-beam computed tomography (CBCT) was performed to assess the three-dimensional morphology and extent of the osseous defect. Radiographic examination demonstrated inadequate bone volume for predictable conventional implant placement, particularly in the facial aspect of the anterior maxilla, with evidence of bone dehiscence. The bone density at the proposed implant region was classified as D3, with a mean radiographic density of approximately 427 HU (Figure 2). This relatively low-density bone indicated a compromised bony substrate and was considered during treatment planning because reduced bone density may influence primary implant stability and the regenerative requirements of the site. However, HU values obtained from CBCT should be interpreted in relation to the imaging system and acquisition parameters rather than as an absolute measure of bone quality. Based on the clinical and radiographic findings, the site was diagnosed as a post-traumatic anterior maxillary ridge deficiency with inadequate bone volume for ideal implant positioning. Guided bone regeneration (GBR), a regenerative technique in which a barrier membrane is used to maintain a protected space for bone formation, was planned simultaneously with implant placement. FDBA was selected as the grafting material to provide a scaffold within the deficient area, while an amnion-chorion membrane was used as a barrier to stabilize the graft and support compartmentalized tissue regeneration. The treatment objective was to achieve adequate peri-implant hard-tissue volume while maintaining the three-dimensional ridge contour required for subsequent prosthetic rehabilitation and esthetic restoration.

**Figure 2:**
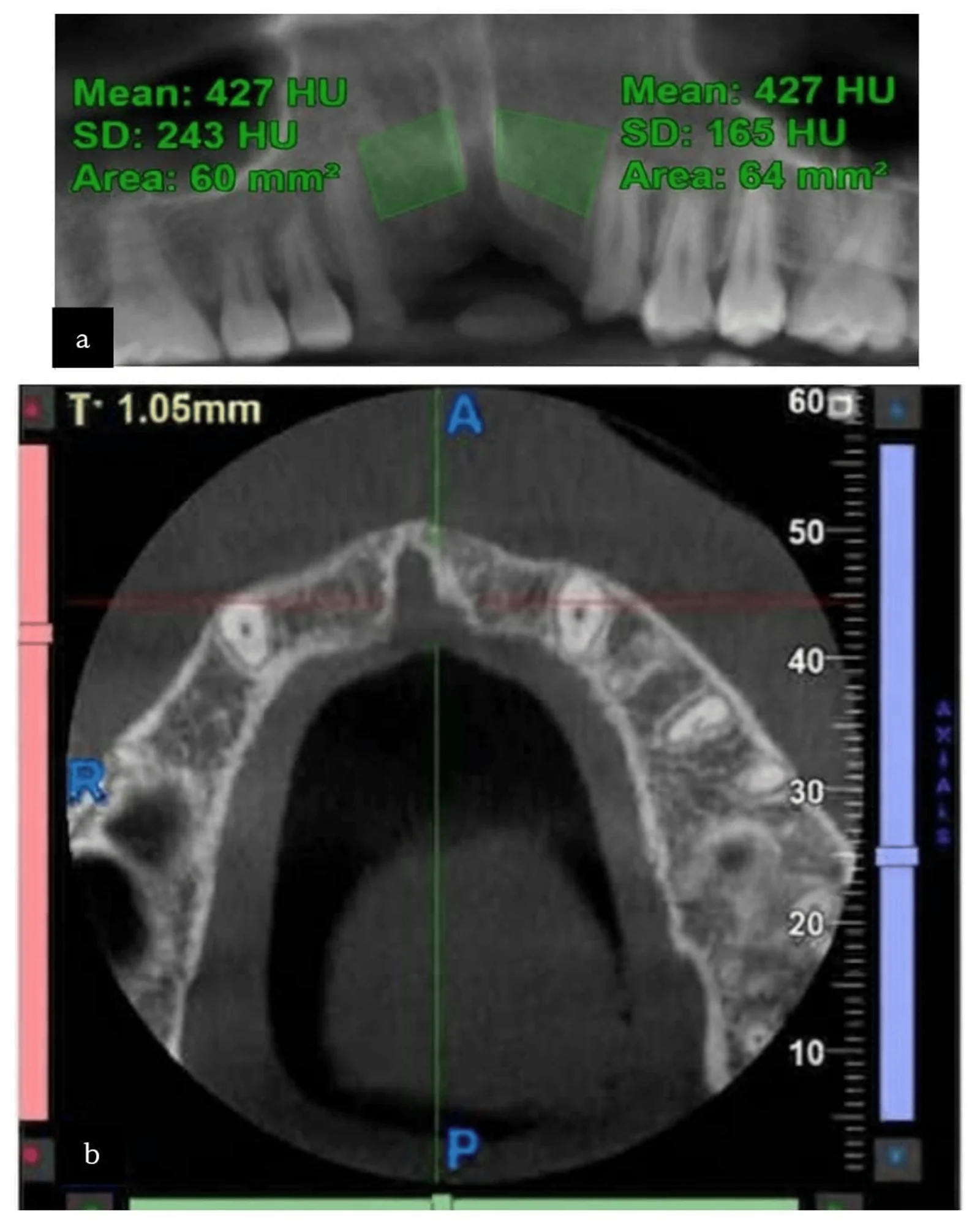
Pre-operative radiographical assessment; a: deficient bone density at the affected region, b: occlusal view showing the bone defect

Under local anaesthesia, a crestal incision was placed, and a full-thickness mucoperiosteal flap was reflected, followed by osteotomy preparation. Two implants, each measuring 3.3 mm in diameter and 11.5 mm in length, were subsequently placed. Primary stability was achieved, with an insertion torque of 37 Ncm. To manage the dehiscence defect and augment the peri-implant bone volume, GBR was performed simultaneously with implant placement. FDBA, obtained from the Tata Memorial Hospital Tissue Bank, Mumbai, was placed within the defect. The grafted site was then covered with a resorbable placental amnion-chorion membrane (also procured from the Tata Memorial Hospital Tissue Bank, Mumbai) to provide a barrier against soft tissue infiltration and facilitate bone regeneration. The surgical flap was repositioned and secured with interrupted sutures using 3-0 non-resorbable silk (Figure 3).

**Figure 3:**
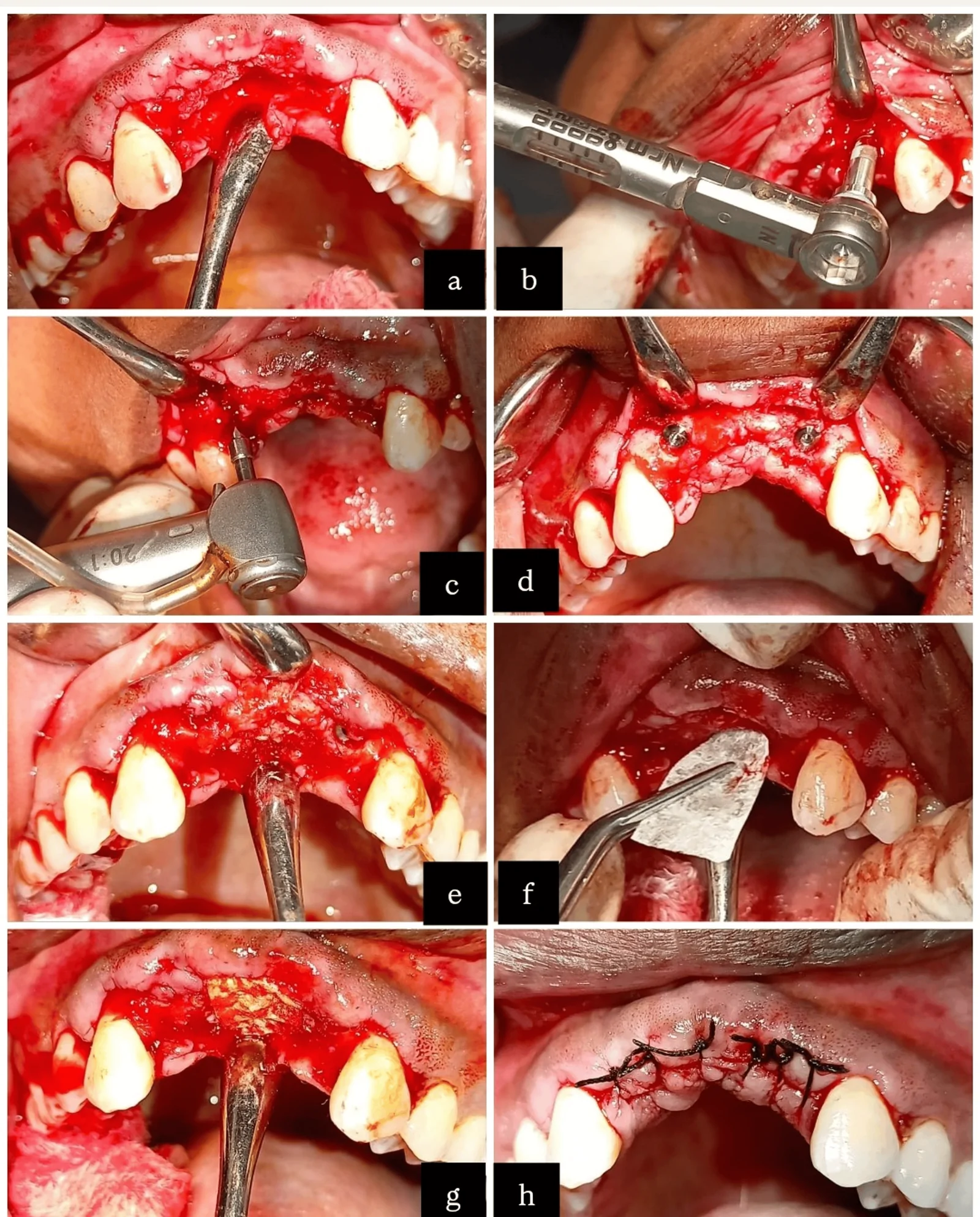
Surgical procedure a: crestal incision is given and bone defect is visible, b,c: implant osteotomy preparation, d: implant placement done, e,f: placement of FDBA bone graft with amnion-chorion membrane, g: graft and membrane secured over the defect, h: flap closure with sutures.

After a healing period of three months, the patient was recalled for phase II implant surgery. Clinical and radiographic evaluation revealed satisfactory healing of the augmented ridge and stable implant placement. Implant uncovering was performed under local anesthesia, followed by placement of appropriate healing abutments to facilitate peri-implant soft tissue conditioning (Figure 4). The surgical site was managed uneventfully, and the patient was advised for follow-up and subsequent prosthetic rehabilitation. At the time of reporting, the implants were clinically stable.

**Figure 4:**
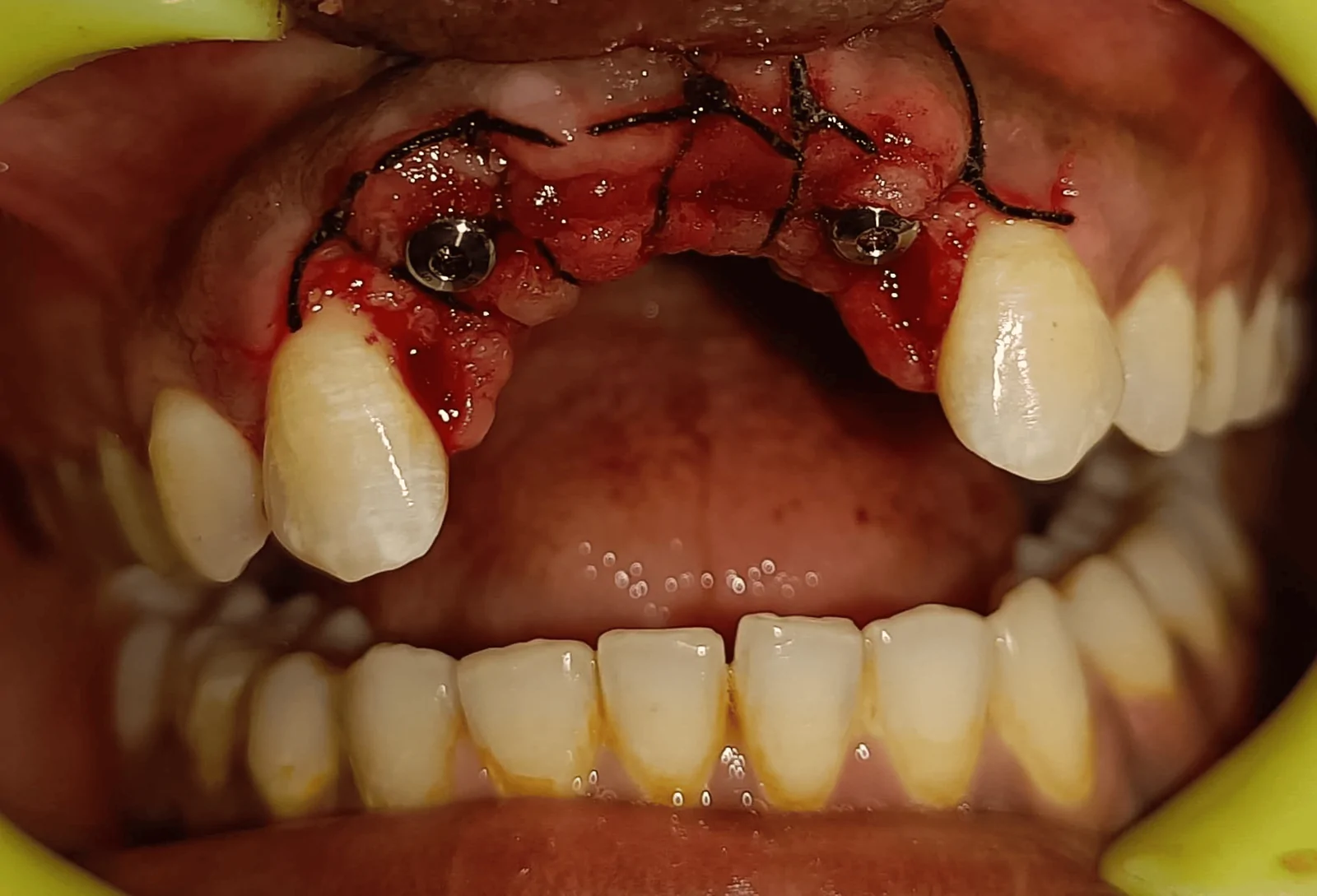
Placement of healing abutments after 3 months

At the six-month follow-up, the surgical site demonstrated satisfactory healing with healthy peri-implant soft tissues and no clinical signs of inflammation or infection. Radiographic evaluation revealed satisfactory bone formation around the implants with maintenance of the augmented bone volume. The implants were clinically stable, indicating successful osseointegration. Following confirmation of satisfactory healing and implant stability, the implants were exposed, and the prosthetic phase of treatment was initiated (Figure 5).

**Figure 5:**
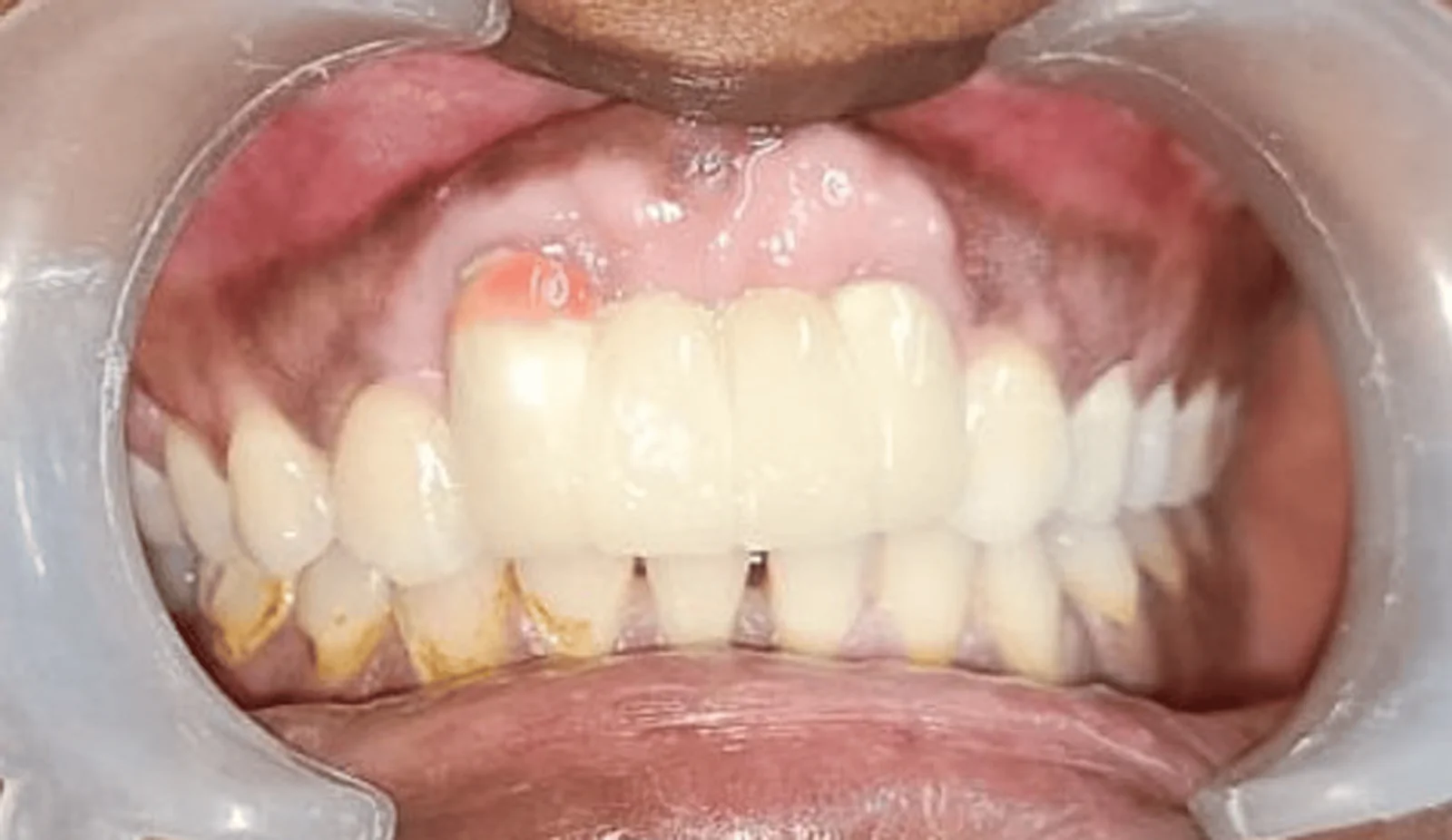
Prosthesis was given after 6 months

At the one-year post-prosthetic follow-up, the implants demonstrated satisfactory clinical and radiographic outcomes. The peri-implant soft tissues appeared healthy and stable, with satisfactory maintenance of the gingival contour and aesthetics. Radiographic evaluation revealed stable crestal bone levels with no significant peri-implant bone loss. The prosthesis remained functionally stable, with no evidence of biological or mechanical complications, indicating successful maintenance of the augmented site and implant-supported rehabilitation (Figure 6).

**Figure 6:**
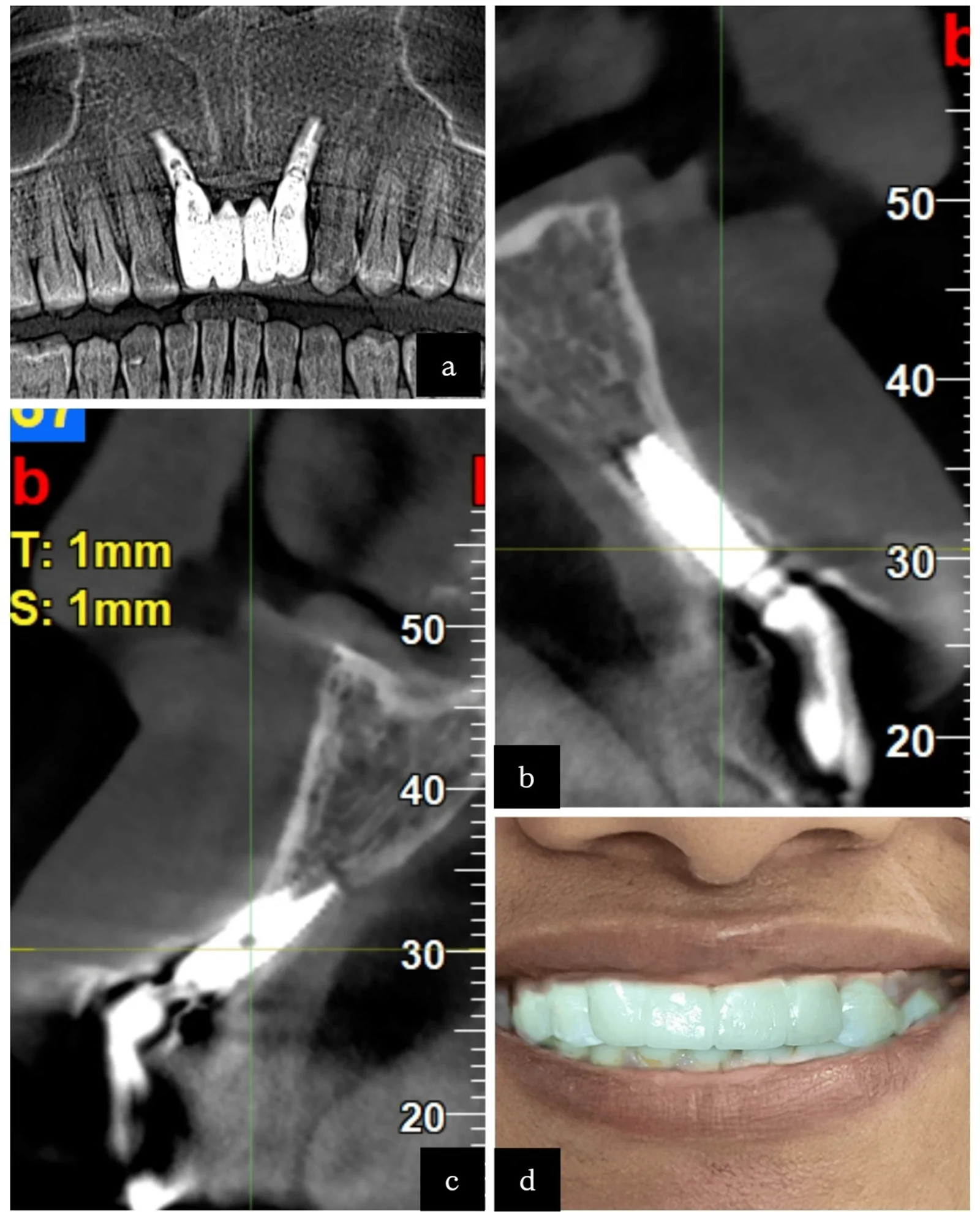
Follow up after 1 year a-c: post operative radiographic evaluation after 1year, d: post operative clinical picture after 1year

Throughout the treatment and follow-up period, the patient reported no complications, such as infection or graft failure. Clinical and radiographic evaluations confirmed stable peri-implant bone levels and healthy soft tissues. This case emphasizes the importance of a combined approach involving initial implant placement along with bone grafting to manage significant bone deficiencies in the upper anterior teeth region, ensuring successful functional and aesthetic outcomes.

## Discussion

The rehabilitation of the maxillary anterior teeth region using dental implants is a complex process that requires careful treatment planning and execution to address functional and aesthetic concerns. This case illustrates the successful use of a simultaneous implant placement and guided bone regeneration approach for ridge augmentation with subsequent implant placement.

GBR is a well-established regenerative procedure that utilizes barrier membranes, often in combination with bone graft materials, to facilitate bone augmentation. The bone graft serves as a scaffold for new bone formation while providing structural support to prevent membrane collapse and maintain the regenerative space. Successful bone regeneration through GBR relies on the selective exclusion of rapidly proliferating epithelial and connective tissue cells, thereby allowing the migration and proliferation of osteogenic and pluripotent cells into the defect site, ultimately promoting new bone formation [6].

In this case, we used FDBA, and its selection as a graft material is well-supported by its osteoconductive properties and its ability to facilitate new bone formation by providing a scaffold for osteoblast attachment and proliferation. FDBA is derived from human donors and undergoes rigorous processing to eliminate immunogenic components while preserving the bone’s natural structure and biological activity [7]. The benefits of FDBA include its slower resorption rate, which enhances its space maintenance properties. Additionally, FDBA is more radiopaque, allowing for better visualization on X-rays compared to the radiolucent demineralized bone allograft [8]. In this case, using FDBA was crucial for augmenting the deficient alveolar ridge, thereby creating a stable foundation for the dental implant.

Previously, non-resorbable membranes like expanded polytetrafluoroethylene have been used and observed to be effective. However, common problems like exposure, inflammation, and compromised bone regeneration have been observed with non-resorbable membranes [6]. Additionally, one important drawback of those non-resorbable membranes is the need for a second surgical procedure for their removal. To overcome these issues, resorbable membranes have been introduced, leading to improved tissue healing, reduced morbidity, quicker resorption, and decreased chances of bacterial contamination [9].

The amnion-chorion membrane is a placental-derived biological membrane that has been investigated as a barrier material in periodontal and implant-related regenerative procedures. Its extracellular matrix is predominantly composed of collagen and contains a range of bioactive components that have been associated with cellular migration, modulation of inflammation, and wound-healing processes. These characteristics provide a biological rationale for its use as a barrier membrane in guided GBR. In the context of implant therapy, the membrane can provide physical separation of the grafted defect from the overlying soft tissues, thereby helping maintain the regenerative space and limiting undesirable soft-tissue infiltration during early healing.

Clinical studies evaluating amnion-chorion membranes in periodontal and implant-related applications have reported their potential utility in soft tissue healing, periodontal regeneration, and management of localized peri-implant defects [10]. However, evidence specifically evaluating amnion-chorion membranes for simultaneous implant placement with GBR remains comparatively limited, and the available clinical studies vary in membrane processing, defect morphology, grafting protocols, and outcome assessment. Therefore, the favorable healing observed in the present case should be interpreted as a clinical outcome of the combined regenerative protocol rather than attributed solely to the membrane.

In the present case, the amnion-chorion membrane was used in conjunction with FDBA during simultaneous implant placement and GBR to cover and stabilize the grafted area. Uneventful soft-tissue healing without membrane exposure or wound dehiscence was observed during the postoperative period. While this outcome supports the clinical feasibility of using an amnion-chorion membrane in the management of an anterior maxillary dehiscence defect, controlled clinical studies with longer follow-up are required to establish its comparative effectiveness for peri-implant bone regeneration.

 Its inherent biocompatibility, anti-inflammatory properties, and ability to act as a biological barrier make it a promising membrane for guided bone regeneration. Unlike conventional barrier membranes, the amnion-chorion membrane is relatively thin and adaptable, allowing close approximation to the surgical site while providing a favorable environment for tissue healing and regeneration.

## Conclusions

The present case demonstrates the successful management of an alveolar bone deficiency in the maxillary anterior region through simultaneous implant placement and GBR. Rehabilitation of this region is particularly challenging due to its high aesthetic demands and the presence of inadequate bone volume following tooth loss. In the present case, implants were placed in the deficient alveolar ridge, followed by augmentation of the peri-implant osseous defect using FDBA covered with a resorbable amnion-chorion membrane. This regenerative approach was employed to augment the deficient ridge, provide a favourable environment for new bone formation, and support long-term implant stability while restoring the peri-implant hard tissue architecture.

## References

[1] Misch CE (2007). Contemporary implant dentistry 3rd edition.

[2] Cucchi A, Chierico A, Fontana F (2019). Statements and recommendations for guided bone regeneration: consensus report of the guided bone regeneration symposium held in Bologna, October 15 to 16, 2016. Implant Dent.

[3] Toledano-Osorio M, Toledano M, Manzano-Moreno FJ, Vallecillo C, Vallecillo-Rivas M, Rodriguez-Archilla A, Osorio R (2021). Alveolar bone ridge augmentation using polymeric membranes: a systematic review and meta-analysis. Polymers (Basel).

[4] Koob TJ, Rennert R, Zabek N (2013). Biological properties of dehydrated human amnion/chorion composite graft: implications for chronic wound healing. Int Wound J.

[5] Giesenhagen B, Martin N, Jung O, Barbeck M (2019). Bone augmentation and simultaneous implant placement with allogenic bone rings and analysis of its purification success. Materials (Basel).

[6] Kolk A, Handschel J, Drescher W (2012). Current trends and future perspectives of bone substitute materials - from space holders to innovative biomaterials. J Craniomaxillofac Surg.

[7] Burchardt H (1983). The biology of bone graft repair. Clin Orthop Relat Res.

[8] Miron RJ (2024). Optimized bone grafting. Periodontol 2000.

[9] Abtahi S, Chen X, Shahabi S, Nasiri N (2023). Resorbable membranes for guided bone regeneration: critical features, potentials, and limitations. ACS Mater Au.

[10] Gulameabasse S, Gindraux F, Catros S, Fricain JC, Fenelon M (2021). Chorion and amnion/chorion membranes in oral and periodontal surgery: A systematic review. J Biomed Mater Res B Appl Biomater.

